# Beyond the Rib Cage: Unraveling Abernethy Syndrome as a Rare Cause of Cyanosis

**DOI:** 10.7759/cureus.60501

**Published:** 2024-05-17

**Authors:** Chaimae Salhi, Chaimae N'joumi, Imane Kamaoui, Maria Rkain, Abdeladim Babakhouya

**Affiliations:** 1 Department of Pediatrics, Mohammed VI University Hospital, Oujda, MAR; 2 Faculty of Medicine and Pharmacy of Oujda, Mohammed Ist University of Oujda, Oujda, MAR; 3 Department of Radiology, Mohammed VI University Hospital, Oujda, MAR; 4 Department of Pediatric Gastroenterology, Centre Hospitalier Universitaire (CHU) Mohammed VI, Oujda, MAR

**Keywords:** malformation, portosystemic shunt, hypoxemia, abernethy, cyanosis

## Abstract

Abernethy syndrome is a rare congenital malformation stemming from a portosystemic shunt. Diagnosis proves challenging due to nonspecific clinical symptoms, with presentation varying based on age and disease severity. Consequences include hepatic, cardiovascular, renal, gastrointestinal, and neurological complications, and growth retardation. We report the case of a child presenting with perioral and digital cyanosis, observed in early childhood. Clinical examination revealed low saturation, telangiectasias, digital clubbing, and collateral venous circulation in the thorax. Imaging confirmed the diagnosis of Abernethy syndrome.

## Introduction

Cyanosis seen in children typically stems from thoracic causes, such as cardiac or pulmonary issues. However, in some cases, cyanosis may arise due to congenital extrahepatic portosystemic shunts (CEPSs) [1]. CEPS refers to a rare condition where there's an abnormal connection between the portal vein system and the systemic circulation, leading to the diversion of portal blood partially or entirely away from the liver [2]. This malformation is estimated to occur in approximately one out of every 30,000 live births [3]. Extrahepatic shunts often occur alongside other congenital anomalies, and they are further categorized into two types according to Morgan and Superina [1]. Abernethy syndrome is a form of CEPS resulting from portal vein agenesis [1]. Diagnosis usually involves Doppler ultrasound of the liver commonly in combination with computed tomography (CT) or magnetic resonance imaging (MRI) of the abdomen [2].

Through our work, we document a case that presented to the Department of Pediatrics of the Mohammed VI University Hospital of Oujda, Oujda, Morocco, for the management of cyanosis. Following thorough evaluation, the diagnosis of Abernethy syndrome was established.

## Case presentation

**Figure 1:**
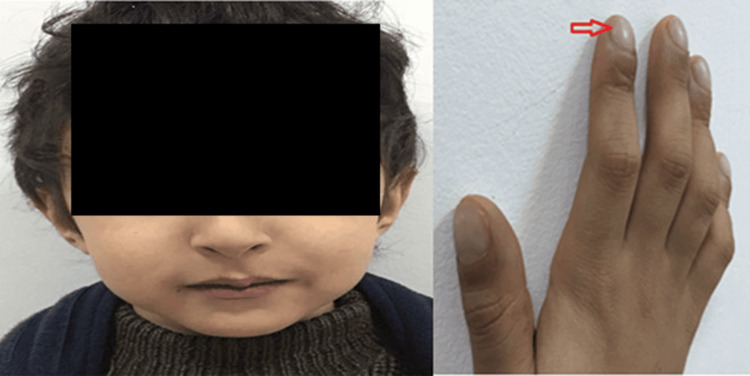
Clinical symptoms of the patient: (A) cyanosis of the lips and (B) digital cyanosis and clubbing.

A five-year-old child, born out of a non-consanguineous marriage, following a full-term pregnancy with normal psychomotor development, was admitted to our department for the management of peribuccal and digital cyanosis. The symptoms started at the age of 2, with cyanosis observed first in the lips and then progressing to the fingers, accompanied by fatigue upon exertion, with no other associated symptoms. The child has never experienced fainting. Upon admission, the clinical examination revealed a conscious child whose vitals were as follows: heart rate of 100 beats/minute, respiratory rate of 18 cycles/minute, blood pressure of 100/65 mmHg, and oxygen saturation of 70% on room air, rising to 85% with supplemental oxygen. He presented with peribuccal cyanosis and digital cyanosis (Figure 1), telangiectasias on the cheeks, and collateral venous circulation in the thorax. Cardiac, pleuropulmonary, and abdominal examinations were unremarkable; notably, no hepatomegaly was observed.

**Figure 2:**
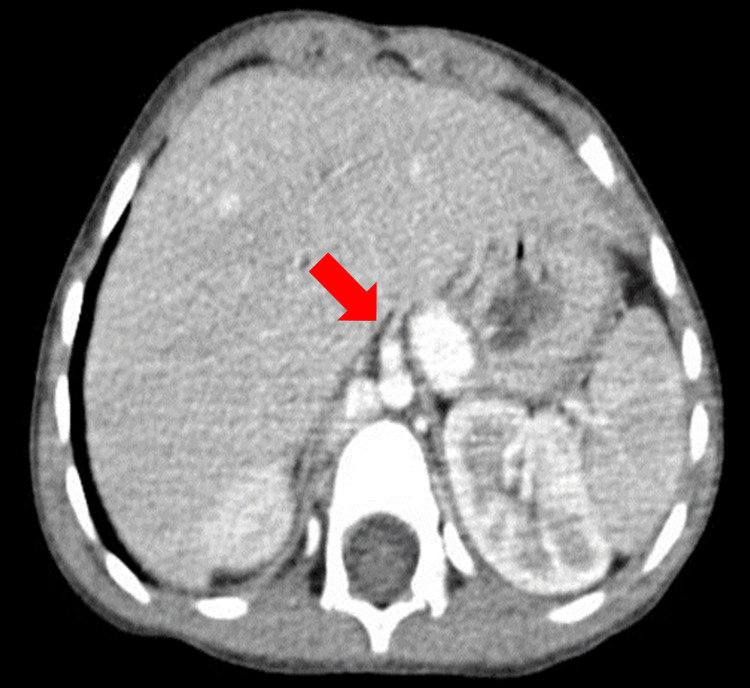
Axial scan section, in the parenchymal window, passing through the liver showing the absence of the portal trunk and its intrahepatic branches.

**Figure 3:**
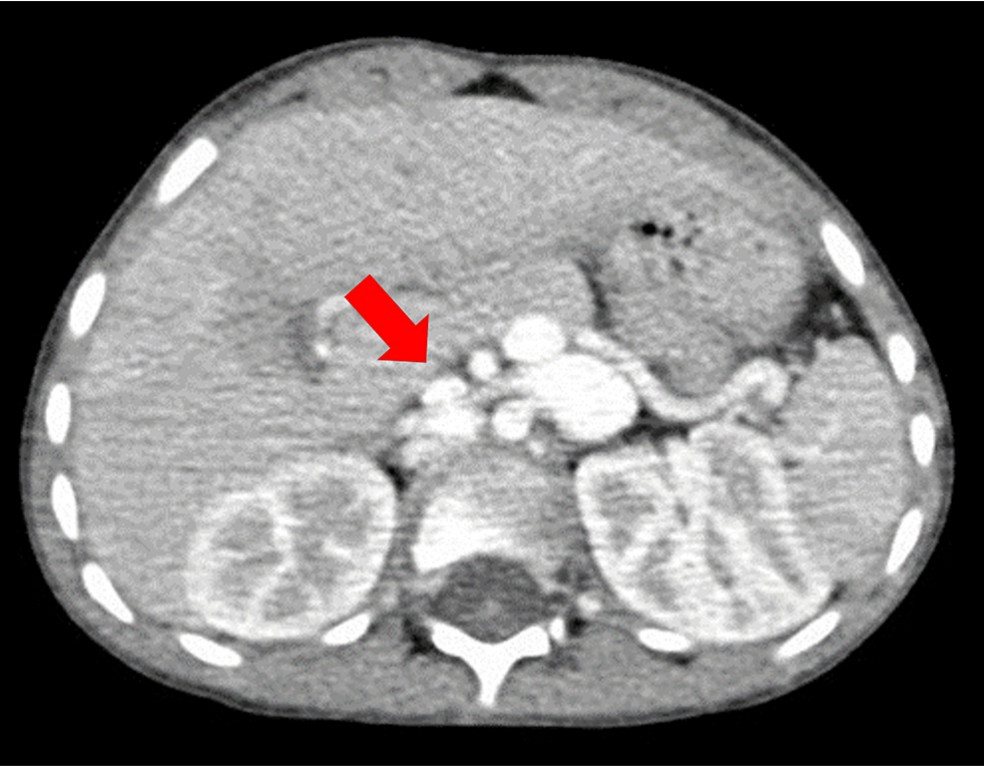
Axial scan section, in the parenchymal window, passing through the liver showing the portosystemic shunt.

Echocardiography was performed on paraclinical assessment, revealing interruption of the inferior vena cava (IVC) with azygos continuation. Abdominal ultrasound showed the absence of the portal vein, prompting further evaluation with thoraco-abdominal CT angiography (Figures 2, 3), which confirmed the absence of the suprarenal and retrohepatic IVC, non-visualized portal system, and a superior mesenteric vein draining into the azygos system to join the superior vena cava. Diagnosis of Abernethy syndrome type I was established. The child's management was initiated abroad, but, unfortunately, he was lost to follow-up.

## Discussion

CEPSs are rare vascular anomalies responsible for abnormal communications between the portal venous system and the systemic venous circulation [1]. They partially or completely bypass the liver's portal perfusion and alter the metabolism of certain metabolites (glucose, galactose, ammonia) [2]. They were first described in 1793 by a London surgeon, John Abernethy, as a communication between the portal vein and the IVC during the autopsy of a 10-month-old infant whose cause of death was unknown [4]. Since then, approximately 80 cases have been reported in medical literature [5].

The main classification system used to diagnose Abernethy malformation was developed by Morgan and Superina [3]. It distinguishes two main types: type I and type II. Type I denotes the complete absence of portal venous branches within the liver, leading to a complete external portovenous shunt to the systemic veins. This category is subdivided into type Ia, where the superior mesenteric vein and the splenic vein drain independently into a systemic vein, and type Ib, where they merge to form a single vein before entering a systemic vein. Conversely, type II of Abernethy malformation is characterized by partial hypoplasia of the intrahepatic portal venous branches, resulting in a partial external portovenous shunt toward the systemic veins [3].

Abernethy syndrome, also known as portal vein agenesis, is a rare cause of hepatopulmonary syndrome, characterized by a congenital extrahepatic systemic shunt [6]. Diagnosis is challenging as clinical symptoms are non-specific; patients may remain asymptomatic or present with hepatic insufficiency, cirrhosis, or isolated cyanosis [5]. The latter may lead to investigations targeting the thoracic cavity, exploring potential pulmonary or cardiac origins.

Abernethy syndrome may be associated with various birth defects (such as heart defects including patent foramen ovale, atrial or ventricular septal defects, patent ductus arteriosus, tetralogy of Fallot, and dextrocardia), hepatic abnormalities (such as hepatic artery enlargement, liver tumors, and biliary atresia), kidney abnormalities (such as cystic renal dysplasia), splenic abnormalities (such as polysplenia), and skeletal abnormalities (notably hemivertebrae and brachydactyly) [4].

The classic triad of hepatopulmonary syndromes consists of liver disease, intrapulmonary vascular dilatation, and arterial hypoxemia [5]. They partially or completely disrupt the liver's portal perfusion and alter the metabolism of certain metabolites such as glucose, galactose, and ammonia [2]. In fact, in cases of CEPSs, high concentrations of galactose may be present in the plasma of newborns without enzymatic deficiencies in galactose metabolism [1].

The advancement of medical imaging has increasingly facilitated the diagnosis of CEPS, thanks to non-invasive investigations such as Doppler echocardiography, CT, or MRI, which allow for classification and thus guide treatment [3].

In terms of treatment, various therapeutic options can be considered. These include early interventional radiology, embolization of the portosystemic shunt, surgical ligation, and, ultimately, liver transplantation as a last resort [6,7]. It is crucial to determine the type of shunt before proceeding with any intervention [3]. Deciding on invasive therapeutic interventions for asymptomatic shunts without associated hepatopulmonary syndrome or encephalopathy remains challenging [4]. According to Tercier et al., asymptomatic shunts may regress; hence, they recommend refraining from therapy until the age of 2 [8]. Hepatopulmonary syndrome caused by Abernethy syndrome is reversible after liver transplantation [9].

## Conclusions

Despite Abernethy syndrome being a rare cause of cyanosis in children, it can lead to serious complications such as hepatic encephalopathy and hepatopulmonary shunt if left untreated. It is important to consider this rare etiology after excluding all cardiac and pulmonary causes. This syndrome, characterized by congenital portosystemic shunts, presents a unique challenge in clinical management and underscores the importance of precise diagnosis and tailored treatment strategies.
